# Modeling sound pulse counting in inferior colliculus

**DOI:** 10.1186/1471-2202-15-S1-P113

**Published:** 2014-07-21

**Authors:** Richard Naud, Dave Houtman, Gary J Rose, André Longtin

**Affiliations:** 1 Department Physics, University of Ottawa, Ottawa, K1N 6N5, Canada; 2 Department Biology, University of Utah, Salt Lake City, UT, 84112, USA

## 

The ability of animals to count events or objects and its underlying mechanisms – including the possible existence of a dedicated “number sense” – is a topic of much recent fascination and research interest. A simple computation that frogs execute routinely is counting the number of consecutive sound pulses in a conspecific call that occur with precise and regular timing [1]. Cells signaling that a threshold number of pulses have occurred have been found in the midbrain of anurans [2]. These counting cells will not respond if a single inter-pulse interval is a few milliseconds longer than the baseline interval. What intrinsic or network mechanisms can give rise to such pulse/interval counting? Comparing simplified neuron models with previously published in vivo membrane potential recordings [3], we identify biophysical processes that can explain the observations. First, we consider a model of phasic inhibition made of onset and offset inhibition. Phasic inhibition enhances reset because a longer interval will engender onset and possibly offset inhibition. Second, we consider four mechanisms, namely short-term facilitation of excitation, persistent sodium currents, dendritic NMDA synapses and recurrent connections of cells imbedded in a network. Combining phasic inhibition with either of these mechanisms can qualitatively reproduce the array of recordings for different pulse patterns – including those with pauses that reset the counting – as well as the effect of pharmacologically attenuating inhibition. These results support the hypothesis that prior segmentation of sound via phasic on and off responses underlies the emergence of features such as pulse counting and duration selectivity in the auditory midbrain.

**Figure 1:**
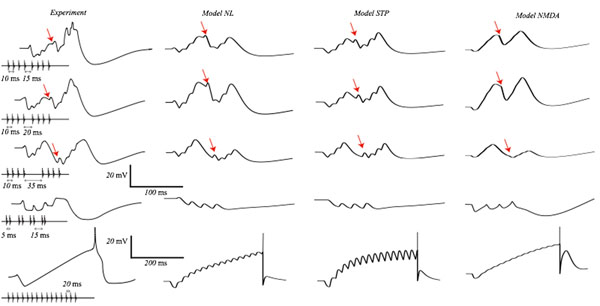
Membrane potential response of counting neurons to series of sound pulses. From left to right columns correspond to: experiments, the persistent sodium model, the short-term plasticity model and the dendritic NMDA model.

## References

[B1] Klump GM Gerhardt HC Use of non-arbitrary acoustic criteria in mate choice by female gray tree frogs Nature 1987 15 286 288 10.1038/326286a0

[B2] Edwards CJ Alder TB Rose GJ Auditory midbrain neurons that count Nat Neurosci 2002 15 10 934 936 10.1038/nn916 12219094

[B3] Edwards CJ Leary CJ Rose GJ Counting on inhibition and rate-dependent excitation in the auditory system J Neurosci 2007 15 49 13384 13392 10.1523/JNEUROSCI.2816-07.2007 18057196 PMC6673096

